# Taxonomy and Phylogeny of Hyphomycetous Muriform Conidial Taxa from the Tibetan Plateau, China

**DOI:** 10.3390/jof9050560

**Published:** 2023-05-12

**Authors:** Rong-Ju Xu, Ying-An Zhu, Ning-Guo Liu, Saranyaphat Boonmee, De-Qun Zhou, Qi Zhao

**Affiliations:** 1 School of Science, Mae Fah Luang University, Chiang Rai 57100, Thailand; xurongju1005@outlook.com; 2 Center of Excellence in Fungal Research, Mae Fah Luang University, Chiang Rai 57100, Thailand; 3 Yunnan Key Laboratory of Fungal Diversity and Green Development, Key Laboratory for Plant Diversity and Biogeography of East Asia, Kunming Institute of Botany, Chinese Academy of Sciences, Kunming 650201, China; 4 College of Landscape and Horticulture, Yunnan Agricultural University, Kunming 650201, China; 5 Faculty of Agriculture, Natural Resources and Environment, Naresuan University, Phitsanulok 65000, Thailand; 6 Academy of Fanjing Mountain National Park, Tongren University, Tongren 554300, China

**Keywords:** freshwater fungi, *Paramonodictys*, *Pleopunctum*, hyphomycetes, Pleosporales

## Abstract

During the investigation of lignicolous freshwater fungi in the Tibetan Plateau habitat, fifteen collections were isolated from submerged decaying wood. Fungal characteristics are commonly found as punctiform or powdery colonies with dark pigmented and muriform conidia. Multigene phylogenetic analyses of combined ITS, LSU, SSU and *TEF* DNA sequences showed that they belong to three families in Pleosporales. Among them, *Paramonodictys dispersa*, *Pleopunctum megalosporum*, *Pl. multicellularum* and *Pl. rotundatum* are established as new species. *Paradictyoarthrinium hydei*, *Pleopunctum ellipsoideum* and *Pl. pseudoellipsoideum* are reported as new records on the freshwater habitats in Tibetan Plateau, China. The morphological descriptions and illustrations of the new collections are provided.

## 1. Introduction

The Tibetan Plateau locates in Central Asia with a mean elevation of more than 4000 m above sea level and an area of about 2,300,000 km^2^ [1]. The Tibetan Plateau encompasses remarkable endemic biodiversity as one of the largest and most unique geographical units on earth [2,3,4,5]. The Tibetan Plateau is rich in water resources, from which almost all major rivers in Asia originate and these rivers serve nearly 1.4 billion people [6]. Furthermore, the Tibetan Plateau, acting as an environmental and ecological barrier [7], has an important effect on ecological security in Asia [8]. It is called the “third pole” of the world [9]. Therefore, it is important to investigate and study the Tibetan Plateau.

Freshwater fungi are a critical component of aquatic ecosystems, playing key roles in the cycling of carbon, nutrients and, energy and are involved in the decomposition of dead organic matter [10,11,12,13]. Despite their ecological importance, freshwater fungi have been often overlooked in studies of freshwater ecosystems [10,12]. The unique environmental conditions of the Tibetan Plateau provide an ideal and unmatched setting for the diversity and distribution of freshwater fungi. The plateau’s high altitude, cold temperatures, and low precipitation create a distinctive set of environmental conditions that shape the uniqueness of the fungal diversity communities and their interactions with other organisms [7,14].

Pleosporales is one of largest orders in Dothideomycetes comprising 94 families, with more than 400 genera [15,16,17,18,19,20]. Parabambusicolaceae was introduced by Tanaka et al. [21], with the generic type *Parabambusicola*. Currently, this family covers 12 genera, including *Aquastroma*, *Lonicericola*, *Multilocularia*, *Multiseptospora*, *Neoaquastroma*, *Neomultiseptospora*, *Parabambusicola*, *Paramonodictys*, *Paramultiseptospora*, *Paratrimmatostroma*, *Pseudomonodictys* and *Scolecohyalosporium* [19,22]. *Paramonodictys* was first introduced by Hyde et al. [23] with *P. solitarius* as the type species, which is saprobic on decaying wood in terrestrial habitats. *Paramonodictys* is characterized by superficial subcylindrical or truncated-cone-form stromata; absent conidiophores; monoblastic conidiogenous cells; muriform, globose or subglobose; dark-pigmented conidia [23]. *Paramonodictys* species occur on decaying wood in both freshwater and terrestrial habitats [20,23].

Paradictyoarthriniaceae was introduced by Liu et al. [24], accommodating an asexual morph genus *Paradictyoarthrinium* based on its unique morphology and distinct lineage in the phylogenetic analysis. Subsequently, *Xenomassariosphaeria*, a sexual morph genus in this family, was established by Wanasinghe et al. [25]. To date, two genera have been accepted in the family *viz*. *Paradictyoarthrinium* and *Xenomassariosphaeria* [19,24,25]. *Paradictyoarthrinium* is characterized by superficial, gregarious, black, powdery and macronematous conidiophores with unevenly dictyoseptate, subglobose to ellipsoidal, dark brown conidia [26]. *Paradictyoarthrinium* was initially established as a monotypic genus, with *Pd. diffractum* as the type species [27]. Subsequently, species of this genus have been continuously described and illustrated [24,26,28,29,30]. So far, four species have been accepted in *Paradictyoarthrinium*, *viz*. *Pd. aquatica*, *Pd. diffractum*, *Pd. hydei* and *Pd. Tectonicola* [24,26,27], http://www.indexfungorum.org/Names/Names.asp accessed on 6 March 2023.

Phaeoseptaceae was established by Hyde et al. [31] to accommodate three genera: *Lignosphaeria*, *Neolophiostoma* and *Phaeoseptum*. Wanasinghe et al. [32] suggested the classification of Phaeoseptaceae includes five genera: *Decaisnella*, *Lignosphaeria*, *Phaeoseptum*, *Pleopunctum* and *Thyridaria*. The type of genus *Phaeoseptum* was initially introduced by Zhang et al. [33] and is known for its sexual morph [31,33,34,35], http://www.indexfungorum.org/Names/Names.asp, accessed on 6 Match 2023. *Pleopunctum*, the first hyphomycetous genus in Phaeoseptaceae, was introduced by Liu et al. [36], with *Pl. ellipticum* as the type species. The genus is characterized by colonies that are scattered, gregarious and, punctiform; macronematous, cylindrical, branched conidiophores; monoblastic, cylindrical conidiogenous cells; and acrogenous, solitary, muriform conidia, often with a hyaline, elliptical to globose basal cell. Currently, seven species have been accepted, all of which are asexual morphs found in China and Thailand, including *Pl. bauhiniae*, *Pl. clematidis*, *Pl. ellipsoideum*, *Pleopunctum heveae*, *Pl. menglaense*, *Pl. pseudoellipsoideum* and *Pl. thailandicum* [32,35,36,37].

In this study, we studied the lignicolous freshwater fungi of the Tibetan Plateau, China. Seven species were collected with the common features of punctiform colonies and muriform conidia. Although these species are morphologically indistinguishable, they belong to three different families within Pleosporales: Parabambusicolaceae, Paradictyoarthriniaceae, and Phaeoseptaceae, as revealed by multi-gene phylogenetic analyses.

## 2. Materials and Methods

### 2.1. Collection, Morphological Examination and Isolation

Submerged decaying wood samples were collected from freshwater habitats in Hengduan Mountains of the Tibetan Plateau, China. Fresh specimens were studied following the methods of Senanayake et al. [38]. Microscopic structures were examined by using a stereomicroscope (SteREO Discovery.V12, Carl Zeiss Microscopy GmBH, Göttingen, Germany), photographed by using a Nikon ECLIPSE 80i compound microscope fitted with a Nikon DS-Ri2 digital camera, and measured by using the Tarosoft (R) Image Framework program. Illustrated figures were processed by using Adobe Photoshop CS6 version 10.0 software (Adobe Systems, San Jose, CA, USA).

Single spore isolation was performed on potato dextrose agar (PDA) plates following the methods described in Senanayake et al. [38]. Fungal herbarium specimens and axenic living cultures were deposited into the Herbarium of Cryptogams of the Kunming Institute of Botany, Chinese Academy of Sciences (KUN-HKAS) and Kunming Institute of Botany Culture Collection (KUNCC), Kunming, China. The novel species were registered in the Faceoffungi [39] and Index Fungorum databases (Index Fungorum 2023).

### 2.2. DNA Extraction, PCR Amplification and Sequencing

Fresh mycelia scraped from colonies growing on PDA plates were used for DNA extraction by using a total DNA extraction kit according to the manufacturer’s instructions (TOLOBIO Plant Genomic DNA Extraction Kit, Shanghai Co., Ltd., Shanghai, China). Polymerase Chain Reaction (PCR) amplifications were performed by using primer pairs ITS5/ITS4 for internal transcribed spacer rDNA region and covered 5.8S ribosomal (ITS), LR0R/LR5 for the nuclear ribosomal large subunit 28S rDNA gene (LSU), NS1/NS4 for the nuclear ribosomal small subunit 18S rDNA gene (SSU) and *TEF1*-983F/*TEF1*-2218R for *TEF1*, respectively [40,41]. The DNA template was carried out in 25 μL reaction volume containing 21 μL of 1 × Power Taq PCR Master Mix, 1 μL of each primer (10 μL stock) and 2 μL of genomic DNA template. Amplifications were carried out by using the BioTeke GT9612 thermocycler (Beijing City, China). The PCR amplification conditions for ITS, LSU and SSU consisted of initial denaturation at 98 °C for 3 min, followed by 35 cycles of denaturation at 98 °C for 20 s, annealing at 53 °C for 10 s, extension at 72 °C for 20 s and the final extension at 72 °C for 5 min. The PCR amplification conditions for *TEF1* consisted of initial denaturation at 98 °C for 3 min, followed by 35 cycles of denaturation at 98 °C for 20 s, annealing at 64 °C for 10 s, extension at 72 °C for 20 s, final extension at 72 °C for 5 min. PCR products were visualized by using 1% agarose gel electrophoresis and distinct bands were checked in Gel documentation system (Compact Desktop UV Transilluminator analyzer GL-3120). The PCR products were sequenced by Tsingke Company, Beijing, China.

### 2.3. Phylogenetic Analyses

The sequences were blasted to search for related taxa in the GenBank database (http://www.ncbi.nlm.nih.gov/blast/, accessed on 6 Match 2023). Sequences generated from the LSU, *TEF1*, SSU and ITS gene regions were carefully verified before further analysis. The new sequences were submitted to GenBank, and the strain information used in this paper was provided in Table 1 [19,20,36]. Multiple sequence alignments were aligned with MAFFT v.7 [42] http://mafft.cbrc.jp/alignment/server/index.html, accessed on 6 Match 2023, and the dataset was trimmed by TrimAlv.1.3 using the gappyout option [43] http://phylemon.bioinfo.cipf.es/utilities.html, accessed on 6 Match 2023. A combined sequence dataset was performed with the SquenceMatrix v.1.7.8 [44].

**Figure 1:**
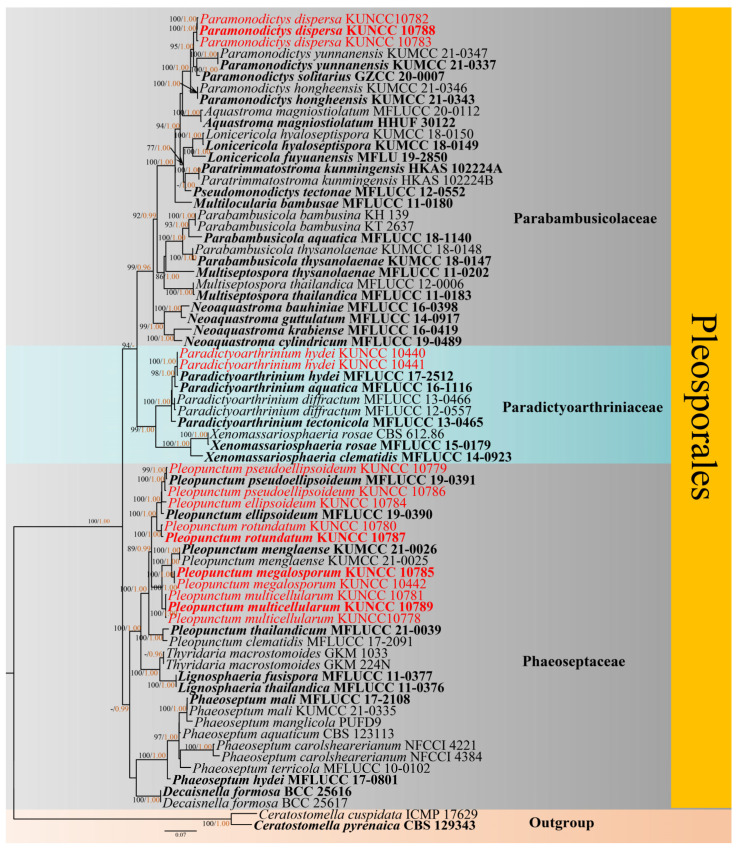
RAxML tree based on analysis of a combined LSU, *TEF1*, SSU and ITS sequence dataset from three families in Pleosporales, *viz*. Parabambusicolaceae, Paradictyoarthriniaceae and Phaeoseptaceae. Bootstrap support values for ML equal to or greater than 75% were given above the nodes (black). PP values equal to or greater than 0.95 were given above the nodes (yellow). The tree was rooted to *Ceratostomella cuspidata* (ICMP 17629) and *C. pyrenaica* (CBS 129343). The type strains were shown in bold, and the newly generated isolates were shown in red.

Maximum likelihood (ML) analysis was performed by RAxML-HPC2 v.8.2.12 [45] in the CIPRES Science Gateway web server [46] http://www.phylo.org/portal2, accessed on 6 Match 2023, by using 1000 rapid bootstrap replicates and the GTRGAMMA+I model. Bootstrap support values for ML equal to or greater than 75% were given above the nodes in the phylogenetic tree (Figure 1).

The model of evolution for the Bayesian inference (BI) analysis was performed by using MrModeltest v2.3 [47]. GTR+I+G was selected as the best-fitting model for LSU, *TEF1*, SSU and ITS dataset. The Markov chain Monte Carlo sampling (BMCMC) was carried out to assess posterior probabilities (PP) by using MrBayes v.3.2.7 [48]. Six simultaneous Markov chains were run for random trees for 1,000,000 generations, and trees were sampled every 200th generation. Bayesian posterior probabilities (PP) equal to or greater than 0.95 were given above the nodes in the phylogenetic tree (Figure 1). Phylograms were visualized by using FigTree v1.4.0 [49] and rearranged in Adobe Photoshop CS6 software (Adobe Systems, USA). The new sequences were deposited in GenBank (Table 1), and the final alignments and phylogenetic tree were registered in TreeBASE under the submission ID: 30133 (http://www.treebase.org/, accessed on 6 Match 2023).

**Table 1:** Taxa used in the phylogenetic analyses and their corresponding GenBank accession numbers.

Species	Isolate No.	GenBank Accession No.				
		ITS	LSU	SSU	*TEF1*-α	References
*Aquastroma magniostiolatum*	MFLUCC 20-0112	MT772005	MT772011	–	MT777678	[50]
***Aquastroma magniostiolatum***	**CBS 139680**	**NR_153583**	**NG_056936**	**NG_061000**	–	[21]
*Ceratostomella cuspidata*	ICMP 17629	KT991671	FJ617558	KT991642	–	[51]
***Ceratostomella pyrenaica***	**CBS 129343**	**KT991672**	**KY931835**	**KY931893**	**–**	[51]
***Decaisnella formosa***	**BCC 25616**	**–**	**GQ925846**	**GQ925833**	**GU479851**	[52]
*Decaisnella formosa*	BCC 25617	–	GQ925847	GQ925834	GU479850	[52]
***Lignosphaeria fusispora***	**MFLUCC 11-0377**	**NR_164233**	**KP888646**	**–**	**–**	[53]
***Lignosphaeria thailandica***	**MFLUCC 11-0376**	**KP899139**	**NG_069268**	**–**	**–**	[53]
***Lonicericola fuyuanensis***	**MFLU 19-2850**	**NR_172419**	**NG_073809**	**NG_070329**	**MN938324**	[54]
***Lonicericola hyaloseptispora***	**KUMCC 18-0149**	**NR_164294**	**NG_066434**	**NG_067680**	**–**	[55]
*Lonicericola hyaloseptispora*	KUMCC 18-0150	MK098194	MK098200	MK098206	MK098210	[55]
***Multilocularia bambusae***	**MFLUCC 11-0180**	**NR_148099**	**NG_059654**	**NG_061229**	**KU705656**	[56]
***Multiseptospora thailandica***	**MFLUCC 11-0183**	**KP744447**	**KP744490**	**KP753955**	**KU705657**	[24]
*Multiseptospora thailandica*	MFLUCC 12-0006	KU693448	KU693441	KU693445	KU705660	[24]
***Multiseptospora thysanolaenae***	**MFLUCC 11-0202**	**–**	**NG_059655**	**NG_063600**	**KU705658**	[56]
***Neoaquastroma bauhiniae***	**MFLUCC 16-0398**	**MH025952**	**MH023319**	**MH023315**	**MH028247**	[57]
***Neoaquastroma cylindricum***	**MFLUCC 19-0489**	**MN473060**	**MN473054**	**MN473048**	**MN481600**	[58]
***Neoaquastroma guttulatum***	**MFLUCC 14-0917**	**KX949739**	**KX949740**	**KX949741**	**KX949742**	[59]
***Neoaquastroma krabiense***	**MFLUCC 16-0419**	**NR_165218**	**NG_067815**	**NG_067670**	**MH028249**	[57]
***Parabambusicola aquatica***	**MFLUCC 18-1140**	**NR_171877**	**NG_073791**	**–**	**–**	[16]
*Parabambusicola bambusina*	KH 139	LC014579	AB807537	AB797247	AB808512	[21]
*Parabambusicola bambusina*	KT 2637	LC014580	AB807538	AB797248	AB808513	[21]
***Parabambusicola thysanolaenae***	**KUMCC 18-0147**	**NR_164044**	**NG_066435**	**NG_067681**	**MK098209**	[55]
*Parabambusicola thysanolaenae*	KUMCC 18-0148	MK098193	MK098198	MK098202	MK098211	[55]
***Paramonodictys dispersa***	**KUNCC 10788**	**ON261165**	**OQ146988**	**OQ135189**	**OQ943185**	This study
*Paramonodictys dispersa*	KUNCC 10782	ON261159	OQ146982	OQ135187	OQ943183	This study
*Paramonodictys dispersa*	KUNCC 10783	ON261160	OQ146983	OQ135188	OQ943184	This study
***Paramonodictys hongheensis***	**KUMCC 21-0343**	**OL436229**	**OL436227**	**OL436232**	**OL505582**	[20]
*Paramonodictys hongheensis*	KUMCC 21-0346	OL436235	OL436224	OL436225	OL505583	[20]
***Paramonodictys solitarius***	**GZCC 20-0007**	**MN901152**	**MN897835**	**MN901118**	**MT023012**	[18]
***Paramonodictys yunnanensis***	**KUMCC 21-0337**	**OL436231**	**OL436226**	**OL436230**	**OL505585**	[20]
*Paramonodictys yunnanensis*	KUMCC 21-0347	OL436233	OL436228	OL436234	OL505586	[20]
***Paratrimmatostroma kunmingensis***	**HKAS 102224A**	**MK098192**	**MK098196**	**MK098204**	**MK098208**	[55]
*Paratrimmatostroma kunmingensis*	HKAS 102224B	MK098195	MK098201	MK098207	–	[55]
***Paradictyoarthrinium aquatica***	**MFLUCC 16-1116**	**MG747496**	**MG747495**	**–**	**–**	[26]
*Paradictyoarthrinium diffractum*	MFLUCC 13-0466	KP744455	KP744498	KP753960	–	[24]
*Paradictyoarthrinium diffractum*	MFLUCC 12-0557	KP744454	KP744497	–	–	[24]
***Paradictyoarthrinium hydei***	**MFLUCC 17-2512**	**MG747498**	**MG747497**	**–**	**–**	[26]
*Paradictyoarthrinium hydei*	KUNCC 10440	OQ135178	OQ146990	OQ135190	OQ943182	This study
*Paradictyoarthrinium hydei*	KUNCC 10441	OQ135179	OQ146991	OQ135191	OQ943181	This study
***Paradictyoarthrinium tectonicola***	**MFLUCC 13-0465**	**KP744456**	**KP744500**	**–**	**–**	[24]
***Phaeoseptum aquaticum***	**CBS 123113**	**KY940803**	**JN644072**	**–**	**–**	[33]
***Phaeoseptum carolshearerianum***	**NFCCI 4221**	**MK307810**	**MK307813**	**MK307816**	**MK309874**	[34]
*Phaeoseptum carolshearerianum*	NFCCI 4384	MK307812	MK307815	MK307818	MK309876	[34]
***Phaeoseptum hydei***	**MFLUCC 17-0801**	**MT240622**	**MT240623**	**MT240624**	**MT241506**	[60]
***Phaeoseptum mali***	**MFLUCC 17-2108**	**MK659580**	**MK625197**	**–**	**MK647990**	[61]
*Phaeoseptum mali*	KUMCC 21-0335	OL413027	OL413028	–	OL690512	[61]
***Phaeoseptum manglicola***	**NFCCI-4666**	**MK307811**	**MK307814**	**MK307817**	**MK309875**	[34]
***Phaeoseptum terricola***	**MFLUCC 10-0102**	**MH105778**	**MH105779**	**NG_065749**	**MH105781**	[31]
***Pleopunctum clematidis***	**MFLUCC 17-2091**	**MT310618**	**MT214573**	**–**	**MT394632**	[35]
***Pleopunctum ellipsoideum***	**MFLUCC 19-0390**	**MK804512**	**MK804517**	**MK804514**	**MK828510**	[36]
*Pleopunctum ellipsoideum*	KUNCC 10784	ON261161	OQ146984	–	OQ943188	This study
***Pleopunctum megalosporum***	**KUNCC 10785**	**ON261162**	**OQ146985**	**–**	**OQ943186**	This study
*Pleopunctum megalosporum*	KUNCC 10442	OQ135180	OQ146986	–	OQ943187	This study
*Pleopunctum menglaense*	KUMCC 21-0025	ON009118	ON009102	ON009086	–	[32]
***Pleopunctum menglaense***	**KUMCC 21-0026**	**ON009119**	**ON009103**	**ON009087**	**–**	[32]
***Pleopunctum multicellularum***	**KUNCC 10789**	**ON261166**	**OQ146989**	**–**	**OQ943190**	This study
*Pleopunctum multicellularum*	KUNCC 10781	ON261158	OQ146981	–	OQ943189	This study
*Pleopunctum multicellularum*	KUNCC 10778	ON261155	OQ146978	–	–	This study
***Pleopunctum pseudoellipsoideum***	**MFLUCC 19-0391**	**MK804513**	**MK804518**	**–**	**MK828511**	[36]
*Pleopunctum pseudoellipsoideum*	KUNCC 10779	ON261156	OQ146979	–	OQ943191	This study
*Pleopunctum pseudoellipsoideum*	KUNCC 10786	ON261163	–	–	OQ943192	This study
***Pleopunctum rotundatum***	**KUNCC 10787**	**ON261164**	**OQ146987**	**–**	**OQ943194**	This study
*Pleopunctum rotundatum*	KUNCC 10780	ON261157	OQ146980	–	OQ943193	This study
***Pleopunctum thailandicum***	**MFLUCC 21-0039**	**MZ198894**	**MZ198896**	**–**	**MZ172461**	[37]
***Pseudomonodictys tectonae***	**MFLUCC 12-0552**	**–**	**NG_059590**	**NG_061213**	**KT285571**	[62]
*Thyridaria macrostomoides*	GKM 1033	–	GU385190	–	GU327776	[53]
*Thyridaria macrostomoides*	GKM 224N	–	GU385191	–	GU327777	[53]
***Xenomassariosphaeria clematidis***	**MFLUCC 14-0923**	**MT310616**	**MT214571**	**–**	**MT394630**	[35]
***Xenomassariosphaeria rosae***	**MFLUCC 15-0179**	**–**	**NG_059883**	**MG829192**	**–**	[25]
*Xenomassariosphaeria rosae*	CBS 612.86	MH862004	MH873692	EF165035		[25]

The newly generated sequences are indicated in red and the ex-type strains are bold and “–” indicated unavailable sequences.

## 3. Results

### 3.1. Phylogenetic Analyses

The concatenated sequence datasets of LSU, *TEF1*, SSU and ITS comprised 71 strains and two outgroup taxa, *Ceratostomella cuspidata* (ICMP 17629) and *C. pyrenaica* (CBS 129343) [51]. The datasets contained 3625 characters including gaps after alignments (LSU: 1–850 bp, *TEF1* = 851–1764 bp, SSU: 1765–2769 bp, ITS: 2770–3625 bp). The RAxML analysis of the combined datasets yielded the best scoring tree with a final ML optimization likelihood value of −24,434.495681. The aligned sequences matrix comprised 1588 distinct alignment patterns with 30.90% of undetermined characters or gaps. Estimated base frequencies were as follows: A = 0.233967, C = 0.259535, G = 0.275768, T = 0.230731, with substitution rates AC = 1.078519, AG = 2.612360, AT = 1.312049, CG = 1.289214, CT = 6.139967, GT = 1.000000; gamma distribution shape parameter α = 0.0010000000. The tree topologies of combined sequence data obtained from ML and BI analyses were not significantly different Figure 1.

The phylogenetic analysis showed that our taxa are distributed in three families i.e., Parabambusicolaceae, Paradictyoarthriniaceae and Phaeoseptaceae into Pleosporales. Three isolates of *Paramonodictys dispersa* (KUNCC 10782, KUNCC 10783 and KUNCC 10788) formed a sister clade with isolates of *Pa. yunnanensis* (KUMCC 21-0337 and KUMCC 21-0347) and *Pa. solitarius* (MFLUCC 17-2353) with 95% ML/1.00 PP support. Two isolates of *Paradictyoarthrinium hydei* (KUNCC 10440 and KUNCC 10441) clustered with the ex-type strain of *Pd. hydei* MFLU 17-2512 (Paradictyoarthriniaceae) with 100% ML/1.00 PP bootstrap support. Ten isolates (KUNCC 10442, KUNCC 10778, KUNCC 10779, KUNCC 10780, KUNCC 10781, KUNCC 10784, KUNCC 10785, KUNCC 10786, KUNCC 10787 and KUNCC 10789) belong to *Pleopunctum* (Phaeoseptaceae) and were identified as two known species, i.e., *Pl. ellipsoideum* and *Pl. pseudoellipsoideum*. The three new species, i.e., *Pl. megalosporum*, *Pl. multicellularum* and *Pl. rotundatum* with high statistical support, are shown in Figure 1.

### 3.2. Taxonomy

**Figure 2:**
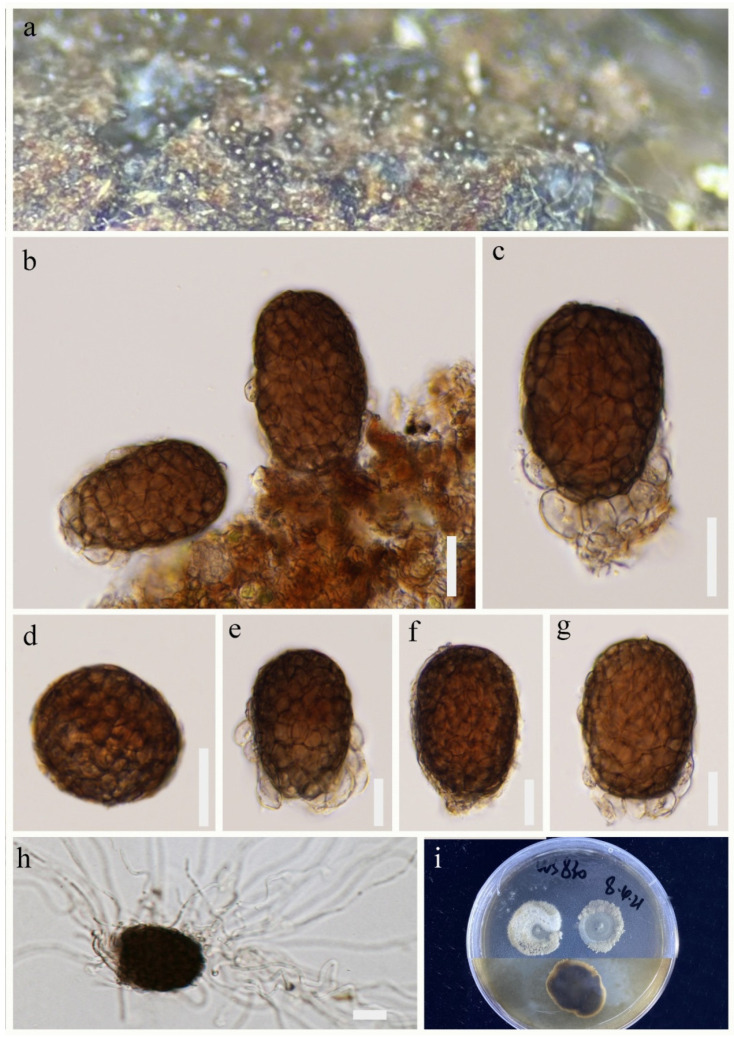
*Paramonodictys dispersa* (HKAS 124267, holotype). (**a**) Scattered, punctiform colonies on natural substrates. (**b**) Conidia on substrate. (**c**–**g**) Conidia with or without multiple hyaline basal cells. (**h**) Germinated conidium. (**i**) Colonies on PDA. Scale bars: (**b**–**h**) = 20 μm.

*Paramonodictys dispersa* R.J. Xu, Q. Zhao and Boonmee, sp. nov., Figure 2.

MycoBank number: MB 847825; Facesoffungi number: FoF 14062

Etymology: Referring to the dispersed colonies.

Holotype: HKAS 124267.

*Saprobic* on decaying wood submerged in freshwater habitats. Sexual morph: Undetermined. Asexual morph: Hyphomycetous. *Colonies* on natural substrate superficial, sporodochial, dark brown to black, scattered, punctiform, glistening. *Mycelium* immersed in the substratum, composed of septate, branched, subhyaline to light brown hyphae. *Conidiophores* micronematous. *Conidiogenous cells* monoblastic, terminal, integrated, light brown. *Conidia* 52–61 × 35–43 μm ($\bar{x}$ = 57 × 38 μm, n = 30), acrogenous, solitary, muriform, irregular septation, subglobose to elliptical, thick-walled, dark brown, basal cells 4–10 × 4–12 μm ($\bar{x}$ = 6 × 7 μm, n = 20), 0–multiple, sometimes extension at the surrounding (Figure 2c), hyaline, elliptical to globose.

Culture characteristics: Conidium germinated on PDA within 48 h. Germ tubes are produced from conidia around. Mycelia superficial, velvet, irregular circular, gray in the central cycle from above, dark brown in the central cycle from below.

Material examined: CHINA, Yunnan Province, Lushui City, Sanhe village (25°55′36″ N, 98°46′9″ E), on submerged wood in a freshwater stream, 30 April 2021, R.J Xu, WS-830 (HKAS 124267, holotype), ex-type living culture, (KUNCC 10788). Furthermore, *ibid*.; (25°55′36″ N, 98°46′9″ E), on submerged wood in a freshwater stream, 30 April 2021, R.J Xu, WS-803 (HKAS 124261), living culture, (KUNCC 10783). Furthermore, *ibid*.; (25°58′9″ N, 98°41′1″ E), on submerged wood in a freshwater stream, 29 April 2021, R.J. Xu, GLG-37 (HKAS 124270), living culture, (KUNCC 10782).

GenBank accession numbers: KUNCC 10788: ITS = ON261165, LSU = OQ146988, SSU = OQ135189, *TEF1*-α = OQ943185. KUNCC 10783: ITS = ON261160, LSU = OQ146983, SSU = OQ135188, *TEF1*-α = OQ943184. KUNCC 10782: ITS = ON261159, LSU = OQ146982, SSU = OQ135187, *TEF1*-α = OQ943183.

Notes: Morphologically, the scattered, punctiform colonies and muriform, globose or subglobose conidia are the typical characteristics in *Paramonodictys*. Simultaneously, *Pa. dispersa* is highly similar to *Pa. hongheensis* (HKAS 122190) [20]. However, *Pa. dispersa* is different from *Pa. hongheensis* by larger conidia (52–61 × 35–43 μm vs. 19–26 × 19–22 µm) [20]. Moreover, *Pa. dispersa* has 0–multiple basal cell extension at the surrounding, while *Pa. hongheensis* has multicellular with regular or more often irregular septation. Phylogenetically, *Pa. dispersa* formed a distinct lineage and was sister to *Pa. solitarius* and *Pa. yunnanensis* with good bootstrap support (95% ML/1.00 PP, Figure 1). However, *P. solitarius* has a globose or subglobose, olivaceous conidia, while *Pa. yunnanensis* has a brownish to reddish-brown, cylindrical conidiogenous cell. Therefore, we dealt with *Pa. dispersa* as a new species based on morphological and phylogenetic analyses.

*Paradictyoarthrinium hydei* N.G. Liu and J.K. Liu, Phytotaxa 338: 290 (2018), Figure 3.

Index Fungorum number: IF554082; Facesoffungi number: FoF 03933

*Saprobic* on decaying wood submerged in freshwater habitats. Sexual morph: Undetermined. Asexual morph: *Colonies* on natural substrate superficial, black, gregarious, powdery. *Mycelium* is mostly immersed, composed of pale brown to dark brown, septate, branched hyphae. *Conidiophores* 2.5–6 μm wide, macronematous, mononematous, short, green, straight to slightly curved, branched, septate, unevenly cylindrical, thick-walled. *Conidiogenous cells* monoblastic, integrated, terminal, determinate, dark green. *Conidia* 14–23 × 10–19 μm ($\bar{x}$ = 17 × 15 μm, n = 25), solitary or catenate, muriform, deeply constricted at the septa, subglobose to ellipsoidal to irregular, green to dark brown in the maturity periods, verrucose.

Culture characters: Conidium germinated on PDA within 24 h. Germ tubes are produced from around. Mycelia circular, gray and dense, with the entire edge. brown to dark brown, black on the reverse.

Material examined: CHINA, Yunnan Province, Lushui City, Gaoligong Mountains, (25°55′36″ N, 98°46′9″ E), on submerged decaying wood in a freshwater stream, 5 May 2021, R.J Xu, XS-31 (HKAS 124272); living culture, (KUNCC 10441). Furthermore, *ibid*.; (25°55′36″ N, 98°46′9″ E), on submerged decaying wood in a freshwater stream, 5 May 2021, R.J Xu, XS-09 (HKAS 124271); living culture, (KUNCC 10440).

GenBank accession numbers: KUNCC 10441: ITS = OQ135179, LSU = OQ146991, SSU = OQ135191, *TEF1*-α = OQ943181. KUNCC 10440: ITS = OQ135178, LSU = OQ146990, SSU = OQ135190, *TEF1*-α = OQ943182.

Notes: *Paradictyoarthrinium hydei* was introduced by Liu et al. [26] from decaying wood in Chiang Mai Province, Thailand. Later Hyde et al. [18] reported *Pd. hydei* on *Quercus variabilis* from Yunnan Province, China. Morphological and phylogenetic analyses showed that *Pd. hydei* (KUNCC 10441) and *Pd. hydei* (KUNCC 10440) clustered with ex-type strain of *Pd. hydei* (MFLUCC 17-2512) with 100% ML/1.00 PP bootstrap support (Figure 1). Therefore, the isolates were identified as *Pd. hydei* and as a new freshwater habitat record to the Tibetan Plateau, China.

*Pleopunctum ellipsoideum* N.G. Liu, K.D. Hyde and J.K. Liu, Mycosphere 10: 767 (2019), Figure 4.

Index Fungorum number: IF 556523; Facesoffungi number: FoF 06114

*Saprobic* on decaying wood submerged in freshwater habitats. Sexual morph: Undetermined. Asexual morph: Hyphomycetous. *Colonies* on wood substrate superficial, sporodochial, brown, scattered, gregarious, punctiform. *Mycelium* immersed in the substratum, composed of septate, branched, subhyaline to grayish brown hyphae. *Conidiophores* macronematous, mononematous, cylindrical, branched, septate, medium brown, smooth and thick-walled. *Conidiogenous cells* monoblastic, terminal, integrated, medium brown. *Conidia* 32–40 × 17–23 μm ($\bar{x}$ = 36 × 19 μm, n = 40), acrogenous, solitary, muriform, constricted at the septa, oval to ellipsoidal, smooth-walled, pale brown when immature, broadly obtuse and dark brown at apex, truncate and paler brown at the base when mature, often with a hyaline, elliptical to globose basal cell, 4–12 × 8–13 μm ($\bar{x}$ = 8 × 9 μm, n = 35).

Culture characteristics: Conidium germinated on PDA within 48 h. Germ tubes are produced from basal cells. Mycelia superficial, velvet, pale in the central cycle and pale gray or white in the outer circle from above. Dark brown to black in the central cycle and dark brown in the outer circle from below.

Material examined: CHINA, Yunnan Province, Lushui City, Dishui River (25°56′3″ N, 98°45′40″ E), on submerged decaying wood in a freshwater river, 28 April 2021, R.J Xu, WS-820 (HKAS 124262), living culture, (KUNCC 10784).

GenBank accession numbers: KUNCC 10784: ITS = ON261161, LSU = OQ146984, *TEF1*-α = OQ943188.

Notes: *Pleopunctum ellipsoideum* was introduced by Liu et al. [36], which is isolated from decaying woods in Guizhou Province, China. Based on phylogenetic analyses, our new isolate KUNCC 21-10784 was placed among two species, i.e., *Pl. ellipsoideum* and *Pl. pseudoellipsoideum* [36] with 100% ML/1.00 PP support (Figure 1). However, our new isolate shares identical morphological characters to the holotype of *Pl. ellipsoideum*. Thus, we identified the two strains as *Pl. ellipsoideum*, and as a new record to the Tibetan Plateau, China.

**Figure 5:**
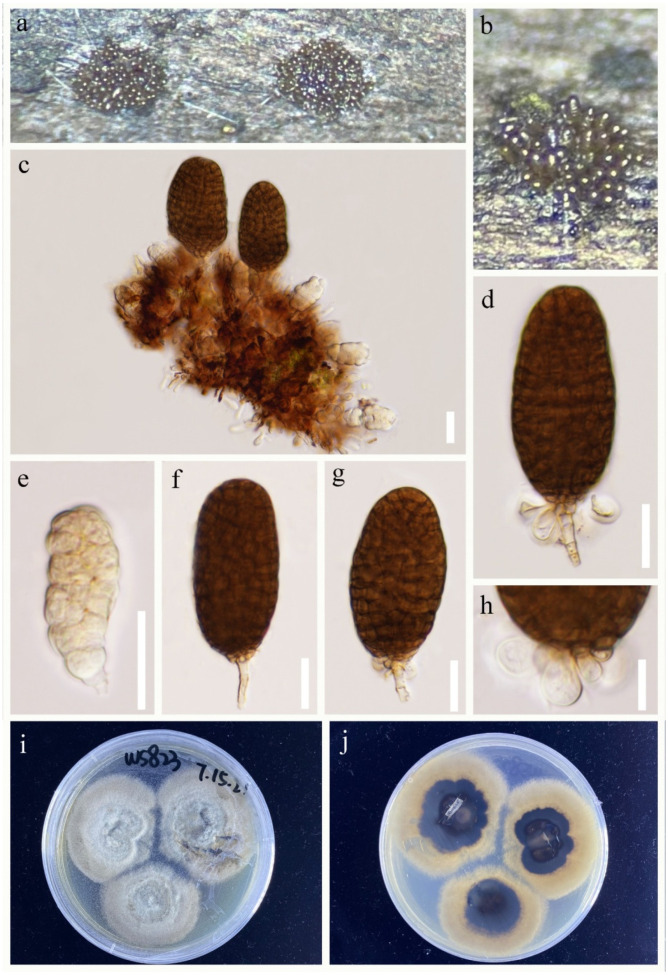
*Pleopunctum megalosporum* (HKAS 124263, holotype). (**a,b**) Colonies on natural substrates. (**c**) Conidia on substrate. (**d**–**g**) Conidiophores and conidia. (**h**) Basal hyaline cells. (**i,j**) Colonies on PDA. Scale bars: (**c**–**g**) = 20 μm, (**h**) = 10 μm.

*Pleopunctum megalosporum* R.J. Xu, Q. Zhao and Boonmee, sp. nov., Figure 5.

MycoBank number: MB 847826; Facesoffungi number: FoF 14063

Etymology: Referring to the macro-conidia.

Holotype: HKAS 124263.

*Saprobic* on decaying wood submerged in freshwater habitats. Sexual morph: Undetermined. Asexual morph: Hyphomycetous. *Colonies* on natural substrate sporodochial, superficial, light brown, scattered, gregarious, punctiform, glistening. *Mycelium* immersed in the substratum, composed of septate, branched, subhyaline to light brown hyphae. *Conidiophores* macronematous, mononematous, cylindrical, unbranched, septate, short, light brown, smooth-walled. *Conidiogenous cells* monoblastic, terminal, integrated, light brown. *Conidia* 51–72 × 28–36 μm ($\bar{x}$ = 63 × 33 μm, n = 40), acrogenous, solitary, muriform, constricted at the septa, oval to long elliptical, smooth-walled, subhyaline to light brown when immature, dark brown when mature, basal cell 10–18 × 7–19 μm ($\bar{x}$ = 14 × 12 μm, n = 20), 0–multiple-basal cells, hyaline, elliptical to globose.

Culture characteristics: Conidium germinated on PDA within 48 h. Germ tubes are produced from basal cell. Mycelia is superficial, velvet, irregular circular, grey in the central cycle from above. Dark brown in the central cycle and yellowish brown in the outer circle from below.

Material examined: CHINA, Yunnan Province, Lushui City, Sanhe village, (25°55′36″ N, 98°46′9″ E), on submerged decaying wood in a freshwater stream, 30 April 2021, R.J Xu, WS-823, (HKAS 124263, holotype), ex-type living culture, (KUNCC 10785). Furthermore, *ibid*.; (25°30′28″ N, 97°55′3″ E) on submerged decaying wood in a freshwater stream, 5 May 2021, R.J Xu, WS-823-1, (HKAS 124264), living culture, (KUNCC 10442).

GenBank accession numbers: KUNCC 10785: ITS = ON261162, LSU = OQ146985, *TEF1*-α = OQ943186. KUNCC 10442: ITS = OQ135180, LSU = OQ146986, *TEF1*-α = OQ943187.

Notes: Phylogenetic analysis shows that *Pleopunctum megalosporum* clustered into a distinctly separated clade and was sister to *Pl. menglaense* with good bootstrap support (100% ML/1.00 PP, Figure 1). However, *Pl. megalosporum* differs from *Pl. menglaense* in having larger conidia (51–72 × 28–36 μm vs. 18–25 × 10–14 µm or 38–55 × 20–26 µm) [32]. Therefore, *Pl. megalosporum* is identified as a new species based on the morphological and phylogenetic analyses.

**Figure 6:**
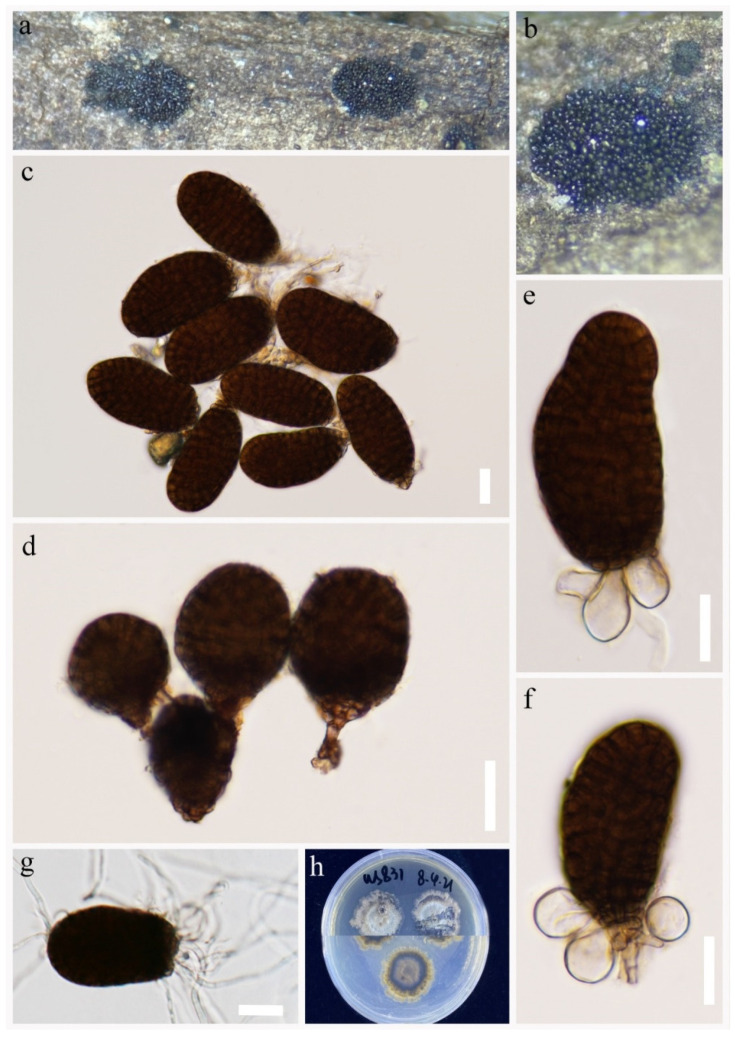
*Pleopunctum multicellularum* (HKAS 124268, holotype). (**a,b**) Colonies on natural substrates. (**c,d**) Conidia mass. (**e,f**) Conidia with hyaline basal cells. (**g**) Germinated conidium. (**h**) Colonies on PDA. Scale bars: (**c**–**g**) = 20 μm.

*Pleopunctum multicellularum* R.J. Xu, Q. Zhao and Boonmee, sp. nov., Figure 6.

MycoBank number: MB 847827; Facesoffungi number: FoF 14064

Etymology: “multicellularum” meaning pluricellular; referring to multiple basal cells.

Holotype: HKAS 124268.

*Saprobic* on decaying wood submerged in freshwater habitats. Sexual morph: Undetermined. Asexual morph: Hyphomycetous. *Colonies* on natural substrate, superficial, dark brown to black, scattered, gregarious, punctiform, glistening. *Mycelium* immersed in the substratum, composed of septate, branched, subhyaline to light brown hyphae. *Conidiophores* 3–5 μm ($\bar{x}$ = 4 μm, n = 20), macronematous, mononematous, cylindrical, unbranched, septate, light brown, smooth-walled. *Conidiogenous cells* monoblastic, terminal, integrated, light brown. *Conidia* 55–71 × 27–39 μm ($\bar{x}$ = 62 × 32 μm, n = 25), acrogenous, solitary, muriform, constricted at the septa, oval to ellipsoidal, smooth-walled, dark brown when mature, broadly obtuse at apex, basal cell 14–20 × 10–17 μm ($\bar{x}$ = 16 × 13 μm, n = 25), 0–3-basal cells, hyaline, elliptical to globose.

Culture characteristics: Conidium germinated on PDA within 48 h. Germ tubes are produced from basal cells. Mycelia superficial, circular, gray from above and pale brown from below.

Material examined: CHINA, Yunnan Province, Lushui City, Gaoligong Mountains, (25°59′4″ N, 98°48′50″ E), on submerged decaying wood in a freshwater stream, 30 April 2021, R.J Xu, WS-831, (HKAS 124268, holotype), ex-type living culture, (KUNCC 10789). Furthermore, *ibid*.; (24°16′8″ N, 101°15′38″ E), on submerged decaying wood in a freshwater stream, 5 May 2021, R.J Xu, ALS-04, (HKAS 124273), living culture, (KUNCC 10778). Furthermore, *ibid*.; (25°58′9″ N, 98°41′1″ E), on submerged decaying wood in a freshwater stream, 30 April 2021, R.J Xu, GLG-29, (HKAS 124269), living culture, (KUNCC 10781).

GenBank accession numbers: KUNCC 10789: ITS = ON261166, LSU = OQ146989, *TEF1*-α = OQ943190. KUNCC 10778: ITS = ON261155, LSU = OQ146978. KUNCC 10781: ITS = ON261158, LSU = OQ146981, *TEF1*-α = OQ943189.

Notes: Morphologically, *Pleopunctum multicellularum* fits well with the generic concept of *Pleopunctum*. However, *Pl. multicellularum* is distinctly different from other species of *Pleopunctum* in having hyaline, 0–3-basal cells and larger conidia. Additionally, phylogenetic analyses show that three *Pl. multicellularum* strains (KUNCC 10781, KUNCC 10789 and KUNCC 10778) clustered into a distinctly separated clade and sharing a sister relationship to *Pl. menglaense* (KUMCC 210026 and KUMCC 210025) and *Pl. megalosporum* (KUNCC 10785 and KUNCC 10442) with good bootstrap support (100% ML/1.00 PP, Figure 1). Therefore, *Pl. multicellularum* is introduced as a new species.

*Pleopunctum pseudoellipsoideum* N.G. Liu, K.D. Hyde and J.K. Liu, Mycosphere 10: 768 (2019), Figure 7.

Index Fungorum number: IF 556524, Facesoffungi number: FoF 06115

*Saprobic* on decaying wood submerged in freshwater habitats. Sexual morph: Undetermined. Asexual morph: Hyphomycetous. *Colonies* on wood substrate superficial, sporodochial, black, gregarious, punctiform. *Mycelium* immersed in the substratum, composed of septate, branched hyphae. *Conidiophores* macronematous, mononematous, cylindrical, septate, medium brown, smooth and thick-walled. *Conidiogenous cells* monoblastic, terminal, integrated, cylindrical, light brown. *Conidia* 36–51 × 22–34 μm ($\bar{x}$ = 44 × 26 μm, n = 25), acrogenous, solitary, muriform, constricted at the septa, oval to ellipsoidal, smooth-walled, broadly obtuse at apex, dark brown, truncate at the base and paler brown, often with hyaline, elliptical to subglobose basal cell, 12–21 × 13–21 μm ($\bar{x}$ = 16 × 16 μm, n = 30).

Culture characteristics: Conidium germinated on PDA within 48 h. Germ tubes are produced from basal cells. Mycelia superficial, irregular circular, flat, entire, dark brown in the center and grayish white near the edge from above. Dark brown in the center with paler towards the edge.

Material examined: CHINA, Yunnan Province, Lushui City, Gaoligong Mountains, (24°16′8″ N, 101°15′38″ E), on submerged decaying wood in a freshwater stream, 5 May 2021, R.J Xu, ALS-14, (HKAS 124274), living cultures, (KUNCC 10779). Furthermore, *ibid*.; (25°56′3″ N, 98°45′40″ E), on submerged decaying wood in a freshwater stream, 5 May 2021, R.J Xu, WS-824, (HKAS 124265), living culture, KUNCC 10786.

GenBank accession numbers: KUNCC 10779: ITS = ON261156, LSU = OQ146979, *TEF1*-α = OQ943191. KUNCC 10786: ITS = ON261163, *TEF1*-α = OQ943192.

Notes: *Pleopunctum pseudoellipsoideum* was isolated from decaying woods in Guizhou Province, China [36]. In a recent study, Wanasinghe et al. [32] described *Pl. pseudoellipsoideum* on decaying wood from Yunnan Province, China. Morphologically, our collections have largely overlapped conidial size with the holotype (36–51 × 22–34 μm vs. 39–59 × 19–28 μm), except for slightly larger basal cell (12–21 × 13–21 μm vs. 6.5–13.5 × 11–15.5 μm) [36]. Furthermore, Phylogenetic analyses provide strong support for this classification, placing the two new strains and the ex-type strain MFLUCC 19-0391 of *Pl. pseudoellipsoideum* in a single clade with 100% ML/1.00 PP support (Figure 1). Therefore, the isolates are identified as *Pl. pseudoellipsoideum*, and they are new record of *Pl. pseudoellipsoideum* in Tibetan Plateau, China.

**Figure 8:**
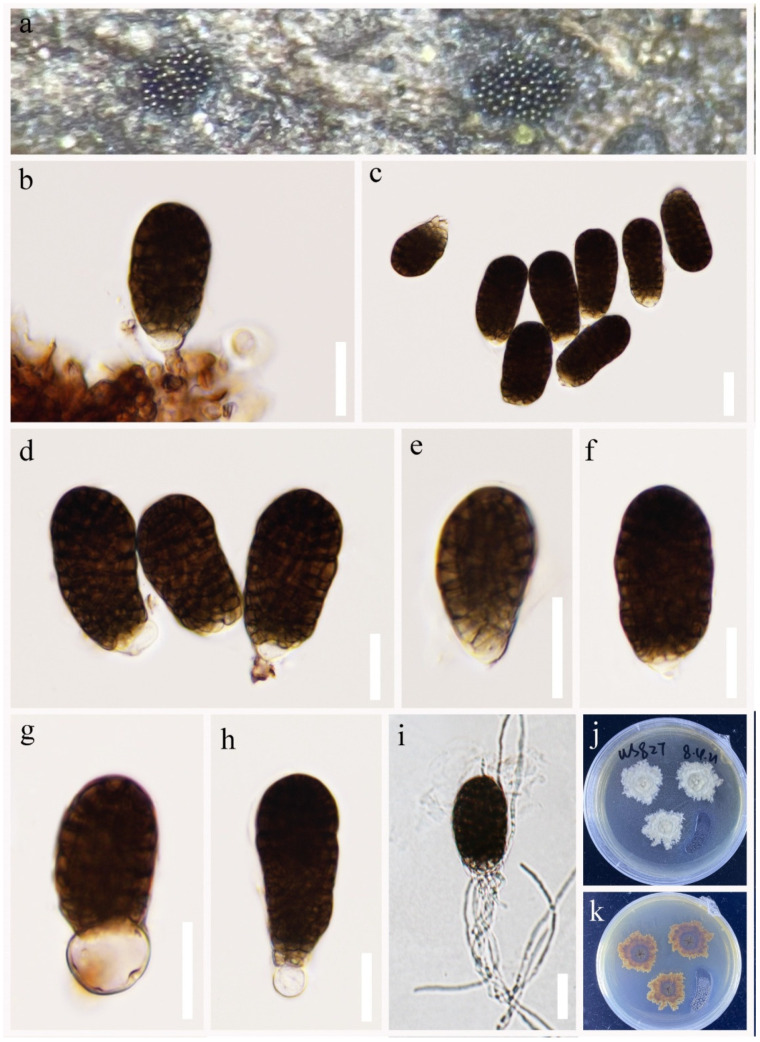
*Pleopunctum rotundatum* (HKAS 124266, holotype). (**a**) Colonies on natural substrates. (**b**) Conidiophores. (**c**–**f**) Conidiogenous cells and conidia. (**g,h**) Conidia with hyaline basal cells. (**i**) Germinated conidium. (**j,k**) Colonies on PDA. Scale bars: (**b**–**i**) = 20 μm.

*Pleopunctum rotundatum* R.J. Xu, Q. Zhao and Boonmee, sp. nov., Figure 8.

MycoBank number: MB 847828; Facesoffungi number: FoF 14065

Etymology: in reference to the rounded base cell.

Holotype: HKAS 124266.

*Saprobic* on decaying wood submerged in freshwater habitats. Sexual morph: Undetermined. Asexual morph: Hyphomycetous. *Colonies* on natural substrate sporodochial, superficial, brown to black, scattered and gregarious, punctiform, glistening. *Mycelium* immersed in the substratum, composed of septate, branched, subhyaline to light brown hyphae. *Conidiophores* are often reduced to conidiogenous cells, short, simple, cylindrical, septate, light brown, smooth-walled. *Conidiogenous cells* monoblastic, terminal, integrated, light brown. *Conidia* 37–52 × 19–27 μm ($\bar{x}$ = 49 × 23 μm, n = 30), acrogenous, solitary, muriform, constricted at septa, oval to ellipsoidal, smooth-walled, broadly obtuse and dark brown at apex, truncate and pale brown at base, basal cell 5–10 × 8–13 μm ($\bar{x}$ = 8 × 11 μm, n = 20), hyaline or light brown, elliptical to globose.

Culture characteristics: Conidium germinated on PDA within 48 h. Germ tubes are produced from basal cells. Mycelia superficial, irregular circular, gray to pale brown in the central cycle from above and pale brown to yellowish to brown from below.

Material examined: CHINA, Yunnan Province, Lushui City, Gaoligong Mountains (25°59′4″ N, 98°48′50″ E), on submerged decaying wood in a freshwater stream, 20 April 2021, R.J Xu, WS-827, (HKAS 124266, holotype), ex-type living culture, (KUNCC 10787). Furthermore, *ibid*.; (25°66′3″ N, 98°52′4″ E), on submerged decaying wood in a freshwater stream, 6 May 2021, R.J Xu, ALS-30, (HKAS 124275), living culture, (KUNCC 10780).

GenBank accession numbers: KUNCC 10787: ITS = ON261164, LSU = OQ146987, *TEF1*-α = OQ943194. KUNCC 10780: ITS = ON261157, LSU = OQ146980, *TEF1*-α = OQ943193.

Notes: *Pleopunctum rotundatum* shares common features in *Pleopunctum* and is similar to *Pl. ellipsoideum* and *Pl. pseudoellipsoideum* in having scattered, gregarious, punctiform, colonies, monoblastic, terminal, integrated conidiogenous cells and acrogenous, solitary, muriform, oval to ellipsoidal conidia. However, *Pl. rotundatum* differs from *Pl. ellipsoideum* in having smaller basal cells (5–10 × 8–13 μm vs. 8–20 × 8.5–18.5 μm), and differs from *Pl. pseudoellipsoideum* in *Pl. pseudoellipsoideum* has a short, septate conidiophores [36]. Furthermore, multi-gene phylogenetic analyses have shown that *Pl. rotundatum* can be classified into a distinct clade, which is the sister group of *Pl. ellipsoideum* and *Pl. pseudoellipsoideum* with high bootstrap support (100% ML/1.00 PP, Figure 1).

## 4. Discussion

The Tibetan Plateau is a region known for its unique biological diversity. Our investigation focuses on the freshwater fungal diversity in the Hengduan Mountains Range, because it is an almost virgin field for the discovery of freshwater fungal diversity. We found seven species from the collections from Hengduan Mountains into Pleosporales based on morphological and multi-gene phylogenetic analyses. The colonies of *Pleopunctum* are gregarious and widely distributed on the substrate [32,35,36,37], while *Paramonodictys* are dispersed and scattered [20,23]. In *Paradictyoarthrinium*, colonies are generally a powdery mass [26]. Thus far, only asexual morphs have been documented in these three genera. Therefore, to gain a more comprehensive understanding of their systematic relationships, it is imperative to obtain further collections and conduct research on their sexual morphs.

The Tibetan Plateau harbors a wide range of aquatic habitats, including lakes, rivers, and wetlands, which support different fungal communities [7]. Although freshwater fungi play an essential role in the ecosystem, they have been understudied in this region due to few researchers have studied freshwater fungi in this region. It is necessary to continue more studies on their diversity, distribution and adaptation to the harsh environmental conditions of the Tibetan Plateau, which has significant implications for our understanding of the functioning in freshwater ecosystems and for the conservation and sustainable use of freshwater resources in this critical region.

## Figures and Tables

**Figure 3:**
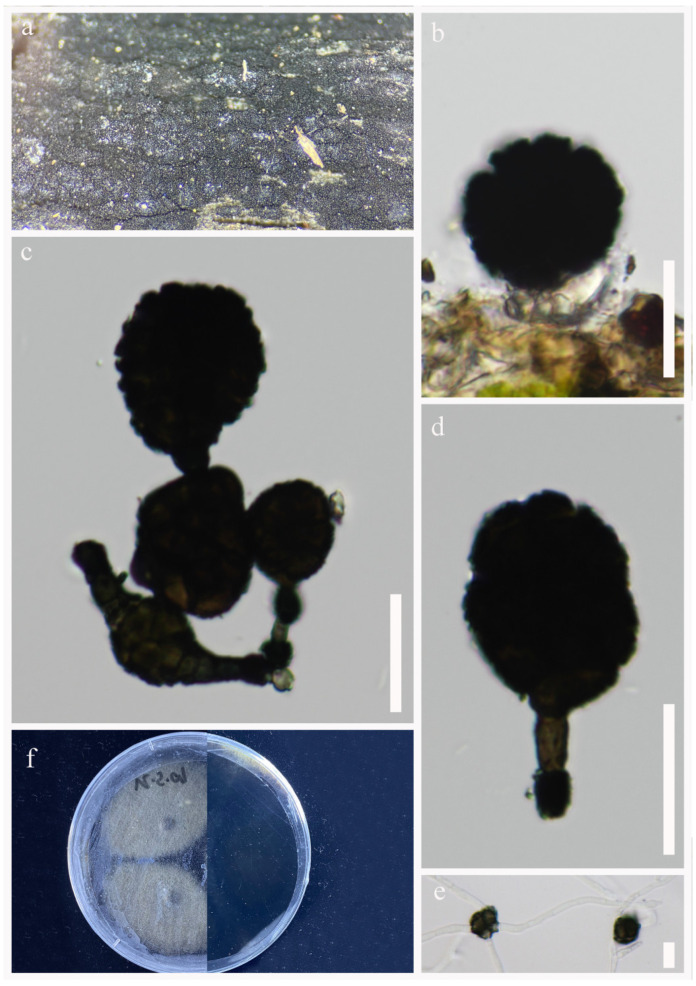
*Paradictyoarthrinium hydei* (HKAS 124272). (**a**) Colonies on natural substrates. (**b**–**d**) Conidiophores and conidia (**e**) Germinated conidia. (**f**) Colonies on PDA. Scale bars: (**b**–**d**) = 20 μm, e = 10 μm.

**Figure 4:**
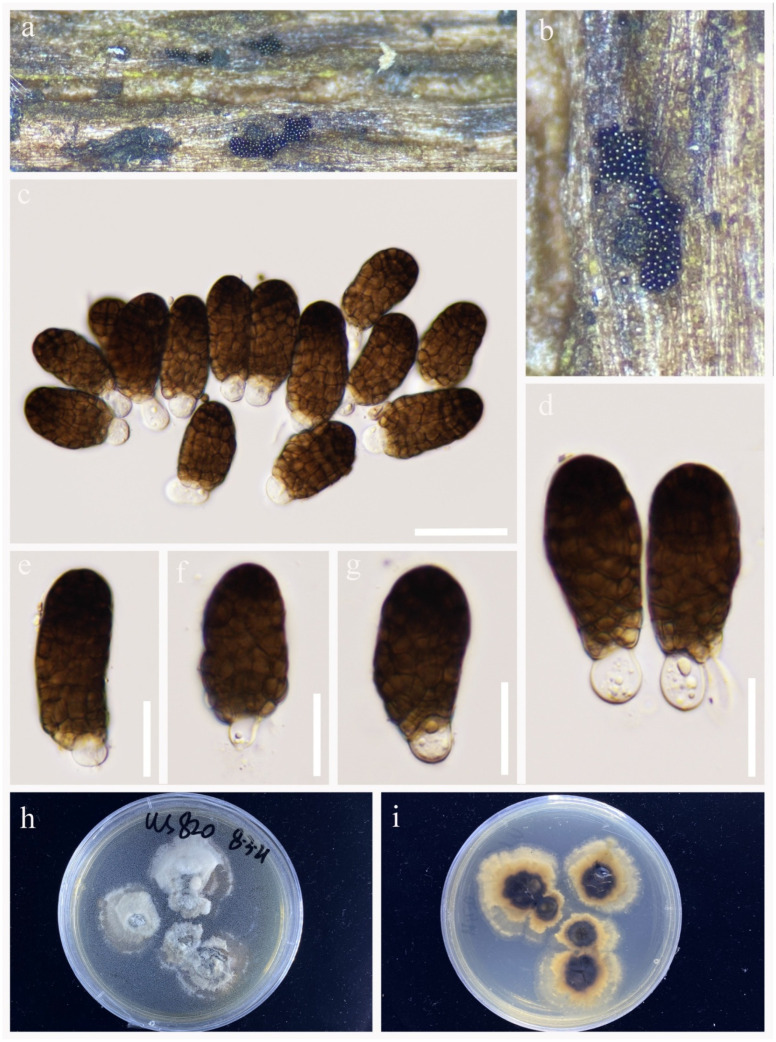
*Pleopunctum ellipsoideum* (HKAS 124262). (**a,b**) Colonies on natural substrates. (**c**–**g**) Conidia with or without basal hyaline cells. (**h,i**) Colonies on PDA. Scale bars: (**c**) = 50 μm, (**d**–**g**) = 20 μm.

**Figure 7:**
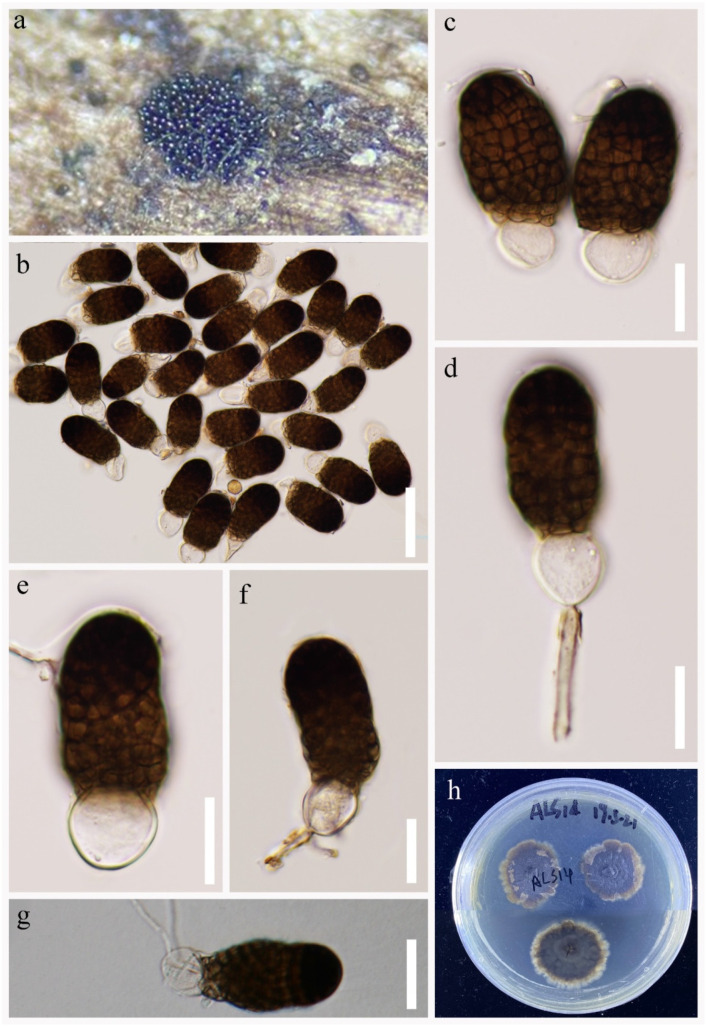
*Pleopunctum pseudoellipsoideum* (HKAS 124274). (**a**) Colonies on natural substrates. (**b,c,e**) Conidia. (**d,f**) Conidiophores and conidia with basal hyaline cells. (**g**) Germinated conidium. (**h**) Colonies on PDA. Scale bars: (**b**) = 50 μm, (**c**–**g**) = 20 μm.

## Data Availability

Not applicable.
